# APOE‐specific Cognitive Effects of Levetiracetam in Mid‐Age Adults

**DOI:** 10.1002/alz70859_100799

**Published:** 2025-12-25

**Authors:** Claire L Lancaster, Nicholas Dowell, Arnold Bakker, Chris Bird, Naji Tabet, Jennifer M Rusted

**Affiliations:** ^1^ Brighton & Sussex Medical School, Brighton United Kingdom; ^2^ Department of Psychiatry and Behavioral Science, Johns Hopkins University School of Medicine, Baltimore, MD USA; ^3^ University of Sussex, Brighton United Kingdom; ^4^ Brighton & Sussex Medical School, Brighton, East Sussex United Kingdom

## Abstract

**Background:**

Low‐dose levetiracetam is under investigation as a potential treatment for slowing the progression of Alzheimer’s Disease (AD). This commonly prescribed anti‐seizure medication has, at low doses, been shown to downregulate excess neuronal activity ‐ a prognostic risk marker observed in the earliest stages of AD, as well as in cognitively healthy carriers of the Apolipoprotein e4 (APOE4+) genetic risk variant. This study tests whether Levetiracetam has acute cognitive benefits in cognitively healthy mid‐age adults, including whether drug effects differ by APOE4 genotype.

**Methods:**

Fifty‐eight adults (aged 45‐65 years old; 27 APOE33; 31 APOE4+) participated in a double‐blind, placebo‐controlled study of low‐dose levetiracetam (125mg bidaily for two‐weeks). At the end of each treatment phase (placebo, levetiracetam), participants completed an executive switch‐inhibition task. Trial‐level data was subject to generalised linear mixed effect modelling to estimate drug by genotype group differences in probability of correct response and response time (RT).

**Results:**

Independent of the effect of treatment, APOE4+ carriers showed poorer accuracy on trials requiring response inhibition (F(1, 15935)=12.39, *p*<.001), and slower RTs across all task trials (F(1, 15107)=12.15, *p*<.001). A significant interaction between drug condition and APOE4 status was reported for both probability of correct response (F(1, 15935)=32.19, *p*<.001) and RT (F1, 15107) = 25.64, *p*<.001). Levetiracetam treatment significantly enhanced performance accuracy (*z‐ratio* = ‐3.05, *p*=.002) and RT (*z‐ratio* = 7.98, *p*<.001) in APOE33 individuals, with greater beneficial effects of Levetiracetam on response speed observed with lower age. Levetiracetam treatment did not significantly affect probability of correct response or RT in APOE4+ individuals (*p*>.05).

**Conclusion:**

Low‐dose levetiracetam selectively enhanced executive function in mid‐age APOE33 individuals, but did not modify performance in APOE4+ carriers, despite literature reporting brain hyperactivity in this at‐risk group. Consistent with existing findings that an 18‐month intervention with low‐dose levetiracetam slows cognitive decline and brain atrophy in non‐APOE4+ individuals with Mild Cognitive Impairment due to AD, this drug may be used prophylactically in those who don’t carry an APOE4 risk variant. Ongoing future work must verify whether Levetiracetam modifies brain hyperactivity in APOE4+ individuals, and if so, what are the longer‐term cognitive and neurological consequences of this intervention.

